# Lupeol, a Pentacyclic Triterpene, Promotes Migration, Wound Closure, and Contractile Effect In Vitro: Possible Involvement of PI3K/Akt and p38/ERK/MAPK Pathways

**DOI:** 10.3390/molecules23112819

**Published:** 2018-10-30

**Authors:** Fernando Pereira Beserra, Meilang Xue, Gabriela Lemos de Azevedo Maia, Ariane Leite Rozza, Cláudia Helena Pellizzon, Christopher John Jackson

**Affiliations:** 1 Department of Morphology, Institute of Biosciences, São Paulo State University (UNESP), Botucatu 18618-689, São Paulo, Brazil; arianerozza@gmail.com (A.L.R.); claudia.pellizzon@gmail.com (C.H.P.); 2 Sutton Research Laboratory, Kolling Institute of Medical Research, the University of Sydney at Royal North Shore Hospital, St Leonard, NSW 2065, Australia; meilang.xue@sydney.edu.au (M.X.); chris.jackson@sydney.edu.au (C.J.J.); 3 Department of Pharmacy, Federal University of São Francisco Valley (UNIVASF), Petrolina 56304-205, Pernambuco, Brazil; gabriela.lam@gmail.com

**Keywords:** lupeol, keratinocytes, fibroblasts, wound healing, cell migration

## Abstract

Skin wound healing is a dynamic and complex process involving several mediators at the cellular and molecular levels. Lupeol, a phytoconstituent belonging to the triterpenes class, is found in several fruit plants and medicinal plants that have been the object of study in the treatment of various diseases, including skin wounds. Various medicinal properties of lupeol have been reported in the literature, including anti-inflammatory, antioxidant, anti-diabetic, and anti-mutagenic effects. We investigated the effects of lupeol (0.1, 1, 10, and 20 μg/mL) on in vitro wound healing assays and signaling mechanisms in human neonatal foreskin keratinocytes and fibroblasts. Results showed that, at high concentrations, Lupeol reduced cell proliferation of both keratinocytes and fibroblasts, but increased in vitro wound healing in keratinocytes and promoted the contraction of dermal fibroblasts in the collagen gel matrix. This triterpene positively regulated matrix metalloproteinase (MMP)-2 and inhibited the NF-κB expression in keratinocytes, suggesting an anti-inflammatory effect. Lupeol also modulated the expression of keratin 16 according to the concentration tested. Additionally, in keratinocytes, lupeol treatment resulted in the activation of Akt, p38, and Tie-2, which are signaling proteins involved in cell proliferation and migration, angiogenesis, and tissue repair. These findings suggest that lupeol has therapeutic potential for accelerating wound healing.

## 1. Introduction

The skin is the largest organ of the human body limiting the organism’s exterior with the external environment whose main functions are protection against external agents, thermoregulation, and perception [1]. The breakdown of skin integrity caused by injury resulting from various events, such as trauma, heat or cold exposure, burns, or blood circulation problems, may render the patient more susceptible to acute or chronic infections, electrolyte imbalance, and fluid loss [2]. In more severe cases, limb amputation may be necessary. Accelerating the wound healing process will assist in circumventing these complications.

Wound healing is a highly complex physiological process involving ordered events classified into three phases that overlap: hemostasis and inflammation (inflammatory phase), granulation tissue formation and re-epithelialization (proliferative phase), and wound contraction and tissue remodeling (extracellular matrix remodeling phase) [3]. This is a natural phenomenon that occurs through cellular and molecular responses and interactions with the main objective of reconstituting and restoring the integrity of the injured tissue [4]. During the cutaneous wound healing process, re-epithelialization via keratinocyte proliferation and migration and (myo)fibroblasts contraction result in wound closure [5].

Medicinal plants have long been reported as a therapeutic alternative for various diseases in many countries. The use of plant extracts or their isolated products for acute and chronic wounds and burns has been well described in the literature [6]. Many of these extracts or plant-derived compounds to treat wounds have pharmacological properties essential for injury repair, such as anti-inflammatory and antioxidant activities [6]. Curcumin, a polyphenol from the rhizomes of *Curcuma longa*, has shown antioxidant properties in several studies for the treatment of many diseases, including skin disorders. Topical applications of curcumin have been widely used when researching cutaneous wound healing. Manca et al. performed in vitro and in vivo studies with curcumin-loaded nanovesicles, which showed antioxidant effects on keratinocytes by reducing oxidative stress and producing an anti-inflammatory effect through decreasing myeloperoxidase activity, edema formation, and providing stimulus for cutaneous re-epithelialization [7]. Quercetin is another well-known natural compound due to its strong antioxidant properties and belongs to the class of flavonoids. A recent study developed from a multiphase system containing quercetin-loaded liposomes showed a significant reduction of lesion area in rats and confirmed its potential in the skin wound treatment [8].

In addition to phenolic compounds and flavonoids, many plant-derived secondary metabolites with antioxidant and anti-inflammatory properties, including the triterpenes, are capable of promoting these healing effects in in vivo and in vitro models [9,10,11]. Triterpenes chemically belong to the class of isoprenoids, which are distributed widely in various parts of the plant from the roots to the fruits. Several studies have shown that triterpenes from medicinal plants improve the quality of healing through mechanisms ranging from the regulation of pro- and anti-inflammatory mediators, chemokines, growth factors, inducing the formation of granulation, re-epithelialization, and wound contraction [12,13,14,15].

*Bowdichia virgilioides* (Fabaceae), is a medicinal plant widely known as “sucupira-preta”, predominantly found in the Brazilian Cerrado in the north, northeast, and central regions of the country. In popular medicine, there are reports of the use of its bark and seeds in the form of an infusion to treat various diseases, such as arthritis, diabetes, bronchitis, and skin wounds [16]. Phytochemical studies revealed that the bark and roots contain a large number of alkaloids and terpenoids, such as lupeol [17,18], volatile constituents and flavonoids [19,20], and anthocyanins [21].

Lupeol is a pentacyclic triterpene found in various species in the plant kingdom, including some vegetables and fruits, such as cucumber, tomato, white cabbage, fig, and guava [22,23]. This molecule exhibits a spectrum of pharmacological activities against various acute or chronic diseases, including arthritis, renal disorders, diabetes, hepatotoxicity, cardiovascular disease, cancer, and microbial infections [24,25,26,27].

In this study, we aimed to explore the effects of the triterpene lupeol on skin wound healing in vitro by investigating proliferation, migration, and cell contraction, as well as its signaling mechanisms involved using human keratinocytes and fibroblasts.

## 2. Results

### 2.1. High Concentrations of Lupeol Decrease Proliferation and Cause Cytotoxicity in Keratinocytes and Fibroblasts

**Figure 1:**
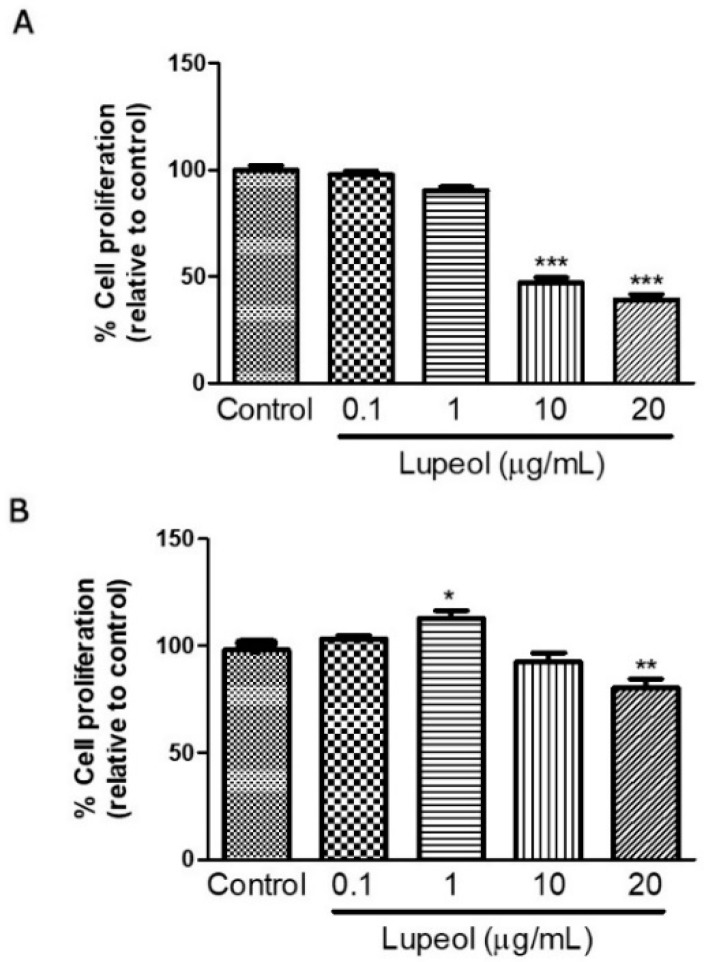
Cell proliferation of (**A**) epidermal keratinocytes and (**B**) dermal fibroblasts in response to lupeol. Cells were seeded in a 96-well plate. After overnight attachment, different concentrations of lupeol were added, and the cells were left for 24 h at 37 °C. Cell proliferation was measured by crystal violet assay and calculated by a comparison of the values from the lupeol treatment group with the control group. Data are expressed as mean ± standard error of the mean (SEM). * *p* < 0.05, ** *p* < 0.01, and *** *p* < 0.001 versus control group.

**Figure 2:**
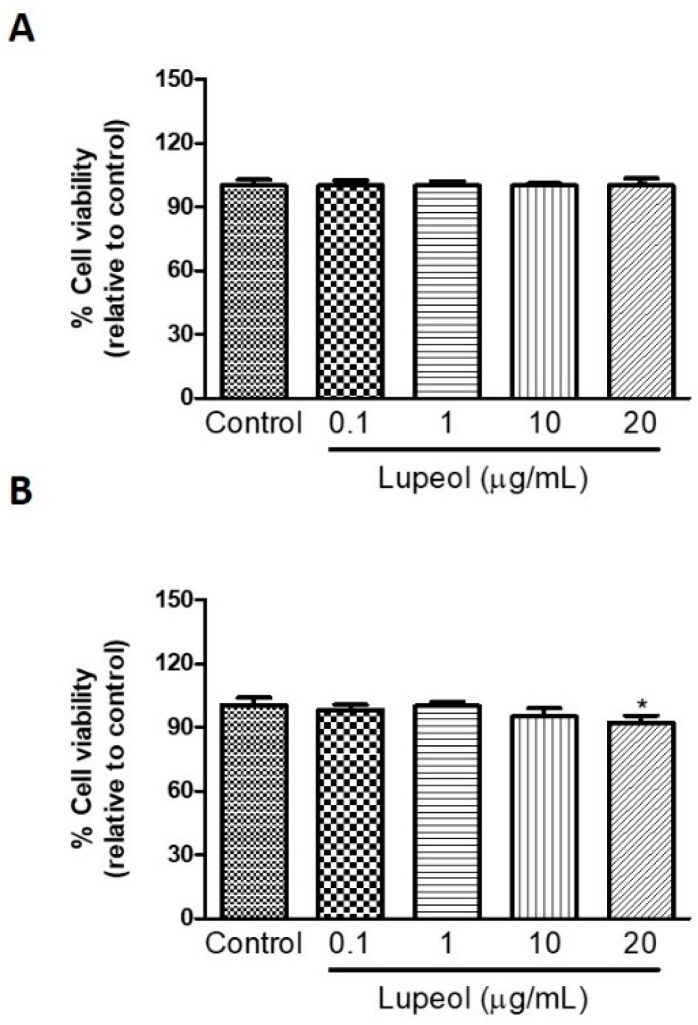
The viability of (**A**) epidermal keratinocytes and (**B**) dermal fibroblasts in response to lupeol. Cells were seeded in a 96-well plate and, after overnight attachment, different concentrations of lupeol were added, and the cells were left for 24 h at 37 °C. Cell viability was measured by MTT assay and calculated by a comparison of the values from the lupeol treatment group with the control group. Data are expressed as mean ± standard error of the mean (SEM). * *p* < 0.05 versus control group.

Human keratinocytes or fibroblasts were treated with lupeol at various concentrations ranging from 0.1 to 20 µg/mL before cell proliferation and viability were assessed by crystal violet and MTT ([3-(4,5-Dimethylthiazol-2-yl)-2,5-Diphenyltetrazolium Bromide]) assay, respectively. The results showed that lupeol at 10 µg/mL and 20 µg/mL, significantly inhibited keratinocyte proliferation after 24 h treatment by 53% and 64%, respectively (Figure 1A). Lupeol at 1 µg/mL increased fibroblast proliferation significantly by 12%, whereas the higher concentration (20 µg/mL) inhibited cell proliferation in relation to the control (19%) (Figure 1B). This triterpene did not affect keratinocyte viability but showed cytotoxicity to fibroblasts at the higher concentration (20 µg/mL) (Figure 2A,B).

### 2.2. Lupeol Enhances Migration and Wound Closure in Human Epidermal Keratinocytes

**Figure 3:**
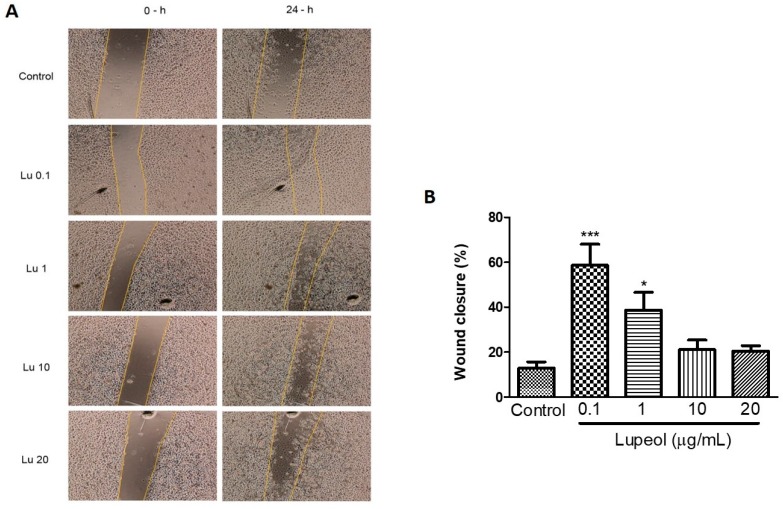
Wound healing effect of lupeol on human epidermal keratinocytes in the scratch assay after 24 h incubation. Cells were incubated with lupeol at concentrations of 0.1, 1, 10, and 20 µg/mL or DMSO at 10 µg/mL as a negative control. (**A**) Representative images of scratch assay at 0 and 24 h. Lu: lupeol. (**B**) Dose-response effect of lupeol on wound closure. Data are expressed as mean ± standard error of the mean (SEM). * *p* < 0.05 and *** *p* < 0.001 versus control group.

The scratch wound healing assay revealed that lupeol (0.1 and 1 μg/mL) significantly increased the wound closure rate compared to the control after 24 h (Figure 3A). The cells treated with the lowest concentration of lupeol, 0.1 μg/mL, showed a 59% increase in the wound closure rate compared to the control (*p* < 0.001). Lupeol at 1 μg/mL also showed a potent wound healing effect on epidermal keratinocytes by 39% (*p* < 0.05). When used at higher concentrations, lupeol did not cause any significant change in wound closure rate.

**Figure 4:**
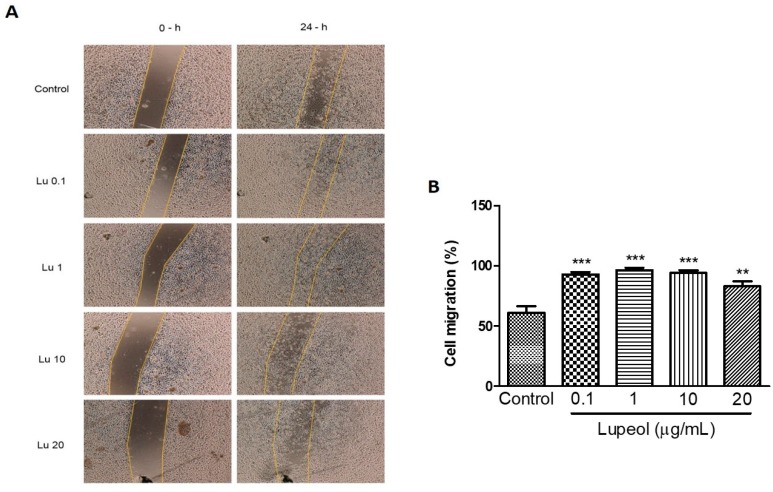
Keratinocyte migration using a scratch assay after 24 h of incubation with lupeol. Cells were incubated with lupeol in different concentrations at 0.1, 1, 10, or 20 µg/mL or DMSO 0.25% at 10 µg/mL as negative control. (**A**) Representative images of scratch assay at 0 and 24 h. Lu: lupeol. (**B**) Effect of lupeol on migration cell. Data are expressed as mean ± standard error of the mean (SEM). ** *p* < 0.01 and *** *p* < 0.001 versus control group.

We further examined whether lupeol stimulates migration in vitro. Scratch migration of human keratinocytes treated with lupeol showed a significant increase in migration at all concentrations tested (0.1 μg/mL 93%, *p* < 0.001; 1 μg/mL 96%, *p* < 0.001; 10 μg/mL 94%, *p* < 0.001; and 20 μg/mL 83%, *p* < 0.05) compared with the control group. Although the higher concentrations were able to inhibit cell proliferation, as shown in Figure 1, these concentrations showed a potent increase in epidermal keratinocytes cell migration (Figure 4B).

### 2.3. Lupeol Promotes Contractile Effect on a Collagen Gel Matrix

**Figure 5:**
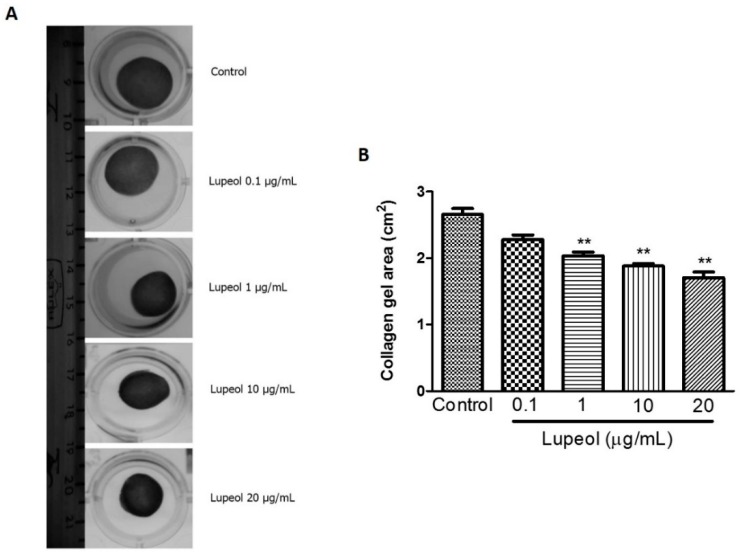
Contractility of lupeol on human fibroblasts. Fibroblasts were cultured within 3D-collagen matrix incubated with lupeol at concentrations of 0.1, 1, 10, or 20 µg/mL or DMSO 0.25% at 10 µg/mL as the negative control. The gels were carefully released from the side wall of the wells and the contractile activity of lupeol was quantified after 48 h of treatment. (**A**) Representative images of collagen matrix at 48 h. (**B**) Semi-quantified results of contractility of lupeol on fibroblasts, expressed as a mean ± SEM. ** *p* < 0.01 versus control group.

To obtain additional information about wound healing mechanisms, we assessed the ability of lupeol to contract a collagen gel matrix containing dermal fibroblasts (Figure 5A). Collagen gel assays provide a convenient platform to investigate a cell’s contractile ability that closely represents cell behavior in vivo. The results showed lupeol significantly increased the contractile effect on collagen gels at 1 µg/mL (*p* < 0.01), 10 (*p* < 0.01), and 20 μg/mL (*p* < 0.01) compared to the control after 48 h of treatment (Figure 5B).

### 2.4. Lupeol Regulates via PI3K/Akt and MAPK Pathways

To identify which signaling pathways are involved in the regulation of human keratinocyte migration promoted by lupeol, we investigated the respective roles of PI3K/Akt, Tie-2, MAPK/ERK/p38, NFκB, and MMP-2 in lupeol-accelerated keratinocyte migration.

**Figure 6:**
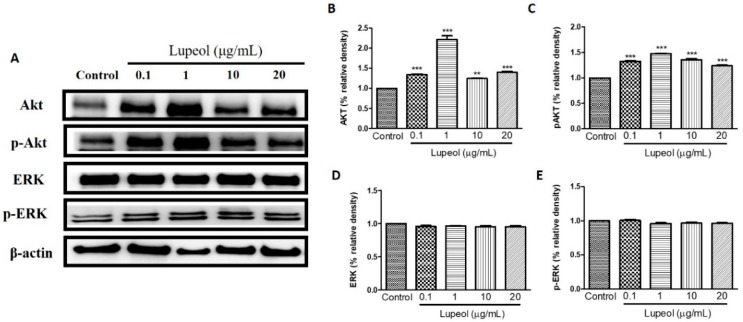
Lupeol regulates the expression of Akt and ERK in epidermal keratinocytes. (**A**) Western blot showing expression of Akt, p-Akt, ERK, and p-ERK in keratinocytes after 24 h treatment with lupeol in different concentrations. (**B**–**E**) All the proteins were semi-quantitated using ImageJ software and normalized to the levels of β-actin. Data are expressed as mean ± SEM. ** *p* < 0.01 and *** *p* < 0.001 versus control group.

As shown in Figure 6A,B, 1 µg/mL lupeol treatment sharply increased the activity of Akt compared with the control (*p* < 0.001). Numerically smaller but significant increases in Akt were observed at all other concentrations (Figure 6B). Lupeol also increased Akt phosphorylation at all concentrations tested (Figure 6C). There was no change in the levels of ERK or p-ERK line response to lupeol (Figure 6D,E).

**Figure 7:**
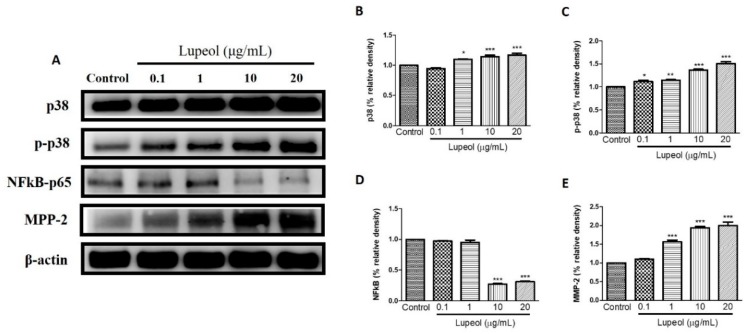
Lupeol regulates the expression of p-38, NF-κB-p65 and MMP-2 in epidermal keratinocytes. (**A**) Western blot showing expression of p38, p-p38, NF-κB-p65, and MMP-2 in keratinocytes after 24 h treatment with lupeol in different concentrations. (**B**–**E**) All the proteins were semi-quantitated using Image J software and normalized to the levels of β-actin. Data are expressed as mean ± SEM. * *p* < 0.05, ** *p* < 0.01, and *** *p* < 0.001 versus control group.

The level of p-p38 and p38 proteins expressed by keratinocytes showed significant changes after treatment with lupeol. A significant increase in p38 was observed in cells treated with lupeol at 1 (*p* < 0.05), 10 (*p* < 0.001) and 20 µg/mL (*p* < 0.001) compared to the control (Figure 7B). p-p38 showed a significant dose-dependent increase in response to all concentrations of lupeol (Figure 7C). MMP-2 activity was significantly enhanced after treatment with 1, 10, and 20 µg/mL of lupeol (Figure 7E). The effect of treatment with lupeol on the expression of pro-inflammatory mediator NFκB was also evaluated. As shown in Figure 7D, lupeol at concentrations of 10 and 20 µg/mL significantly decreased the expression of NFκB compared to the control.

**Figure 8:**
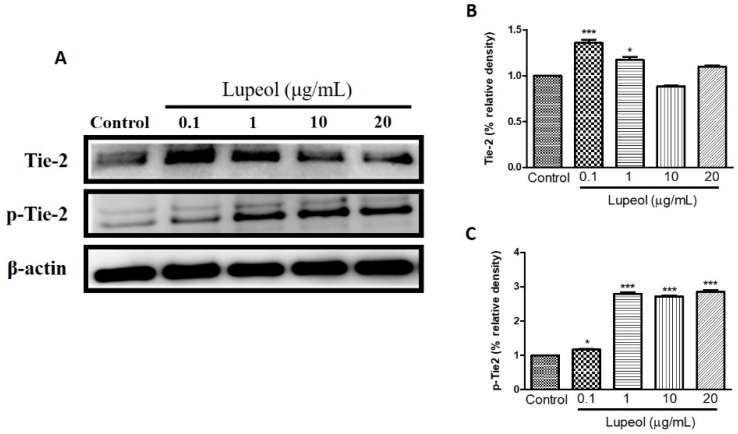
Lupeol regulates the expression of Tie-2 in epidermal keratinocytes. (**A**) Western blot showing expression of Tie-2 and p-Tie-2 in keratinocytes after 24 h treatment with lupeol in different concentrations. (**B**,**C**) All the proteins were semi-quantitated using Image J software and normalized to the levels of β-actin. Data are expressed as mean ± SEM. * *p* < 0.05 and *** *p* < 0.001 versus control group.

We examined the influence of lupeol treatment on Tie-2 expression, a protein-tyrosine kinase receptor expressed by endothelial and epithelial cells, which functions to stabilize the cell barrier. Treatment with lupeol showed a dose-dependent decrease in protein expression of Tie-2, with a concomitant increase in the levels of its phosphorylated isoform, p-Tie-2, clearly indicating that lupeol activates Tie-2 (Figure 8C).

### 2.5. Lupeol Regulates the Differentiation of Cytokeratin 16

**Figure 9:**
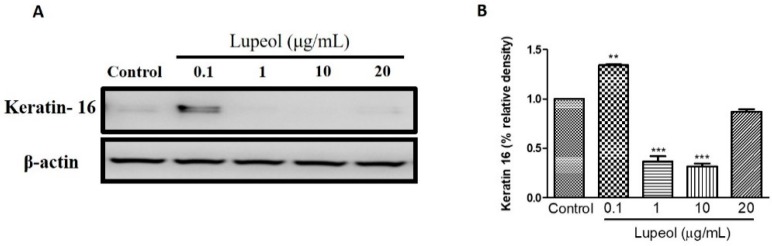
Lupeol regulates the expression of keratin 16 in epidermal keratinocytes. (**A**) Western blot showing expression of keratin 16 in keratinocytes after 24-h treatment with lupeol. (**B**) All the proteins were semi-quantitated using ImageJ software and normalized to the levels of β-actin. Data are expressed as mean ± SEM. ** *p* < 0.01 and *** *p* < 0.001 versus control group.

To understand the effect of lupeol on the differentiation of keratinocytes, we investigated keratin 16 expression in keratinocytes after 24 h treatment. As shown in Figure 9A,B, 0.1 µg/mL lupeol treatment increased keratin 16 compared to control (*p* < 0.001). Significant decreases in keratin 16 were observed with lupeol at 1 and 10 µg/mL, with no change in response to 20 µg/mL.

## 3. Discussion

**Figure 10:**
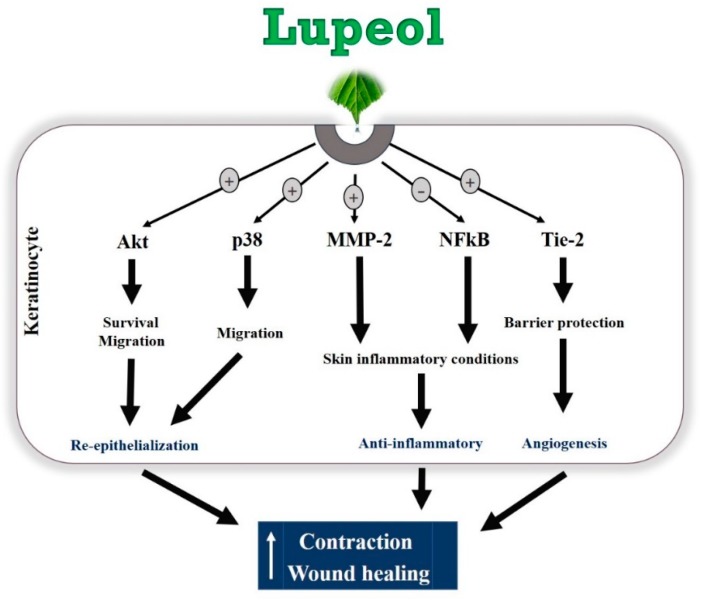
Hypothetical model of the regulatory mechanisms of lupeol in human keratinocytes on wound healing.

Lupeol, a natural compound isolated from *B. virgiliodes,* plays protective roles in a variety of cancers, skin inflammation, pancreatitis, arthritis, diabetes, and hepatic and cardiovascular diseases [28,29,30,31,32,33,34], but little is known about its effects on in vitro wound healing. In this study, we demonstrated that lupeol enhances in vitro wound healing, possibly by stimulating the survival, migration, and contraction of epidermal keratinocytes and/or dermal fibroblasts (Figure 10).

Wound healing is a highly complex biochemical and physiological process involving various cellular and molecular mediators, such as chemokines and cytokines, inflammatory infiltrate, immune system cells, growth factors, and extracellular matrix proteins [2]. Keratinocyte proliferation and migration are crucial events in wound repair. These cells migrate toward the wound surface and fill the wound area to cover the open space exposed to infections, thereby contributing to the wound healing effect [5,35]. Keratinocyte migration may occur independently of keratinocyte proliferation during re-epithelialization [36]. This cell mechanism is associated with the formation and disassembly of cell adhesion sites and cytoskeletal reorganization [35]. Using an in vitro migration assay, which did not involve cell proliferation, we found that lupeol had a strong effect at all concentrations, indicating that lupeol may accelerate cutaneous wound repair by stimulating the migration of keratinocytes. In contrast, we found that lupeol at low concentrations (0.1 and 1 μg/mL) exhibited a potent wound healing effect in vitro, a process in which both proliferation and migration are involved. Our study also demonstrated that lupeol, at high concentrations, inhibited cell proliferation in both cell types and stimulated cytotoxicity in fibroblasts. Taken together, these results suggest that cell migration, rather than cell proliferation, is the more prominent pathway for lupeol to promote cutaneous re-epithelialization and wound repair in keratinocytes.

After re-epithelialization and extracellular matrix reorganization, wound contraction is an important event during the wound healing process. Our collagen gel contraction assay showed that lupeol treatment stimulated the contractile effect of fibroblasts embedded in a collagen gel solution in a dose-dependent manner. This culture system has been widely used as a model for pro-contractile remodeling of the extracellular matrix by dermal fibroblasts for situations such as wound healing [37]. This fibroblast-embedded collagen gels system allows cells to develop endogenous tension through both mechanical and biochemical signals relevant for cell contractility, thereby triggering signal transduction cascades capable of modulating transmembrane and/or intracellular receptors to promote intracellular responses such as gene expression and protein synthesis [38].

It has been reported that activation of the PI3K/Akt pathway triggers mechanisms responsible for cell polarity capable of influencing the migration speed, thus leading to the migratory activity of various cell types, including keratinocytes and fibroblasts [39,40]. In addition, PI3K/Akt is a pathway that has been implicated as an important mediator in the control of survival/cell growth, malignant and metastatic oncogenic transformation, and the regulation of various diseases [41]. In our study, protein expression determined via Western blot clearly showed that lupeol significantly up-regulated Akt expression and activation in epidermal keratinocytes, suggesting that Akt may be a key signaling component in lupeol-induced keratinocyte migration and wound healing.

MAPK signaling is an important pathway formed by a chain of proteins in the cell, which is responsible for modulating the synthesis and release of growth factors involved in cell proliferation and migration during wound healing [42]. The MAPKs consist of extracellular signal-regulated protein kinase (ERK), c-Jun NH2-terminal kinase (JNK), and p38 mitogen-activated protein kinases [43]. ERK and p38 are crucial signaling molecules in wound healing. ERK plays a crucial role in the regulation of cell migration, proliferation, differentiation, and cell survival [44]. Although the role of the p38 MAPK signaling pathway in wound healing has not yet been fully elucidated, recent studies have suggested its involvement in the migration of keratinocytes to wounds [45]. In this study, lupeol did not significantly change ERK expression or activation, but increased the p38 activation in a concentration-dependent manner. As such, we hypothesize that lupeol-induced migration and wound closure in human keratinocytes occurs through activation of p38 MAPK.

Inflammation is a crucial event characterized by the infiltration of inflammatory cells into the injured tissue. Successful tissue regeneration is directly related to resolution of the inflammatory phase, since low recruitment of inflammatory cells is associated with delayed healing, and excessive inflammation can result in chronic wounds, complicating the repair formation of a scar. The NF-κB pathway plays a key role in controlling the expression of various inflammatory genes including TNF-α (Tumor necrosis factor-alpha), cell adhesion molecules such as E-selectin, and vascular cell adhesion molecule-1 [46]. This protein complex is located within the cytoplasm as a pair of dimers (p50/p65). Once activated by inflammatory stimuli, including surgical lesions, NF-κB dimers migrate to the nucleus of the cell binding to the molecules of DNA, thereby inducing the transcription of a variety of genes responsible for proliferation, migration, cell cycling, inhibition of apoptosis, and most importantly, inflammation [47,48]. In our study, high doses of lupeol suppressed the activation and expression of NF-κB in keratinocytes, thus contributing to the dampening of the inflammatory process in cutaneous wounds.

Another interesting finding in this study was that lupeol significantly increased the expression of MMP-2 in a concentration-dependent manner in human keratinocytes. MMPs present in keratinocytes are enzymes involved not only in the modulation of inflammation but also in the proliferative phase, in cell proliferation/migration control, and contribute to skin barrier function and extracellular matrix remodeling [49,50]. MMP-2, in particular, has strong anti-inflammatory properties [50]. Our findings, which showed that lupeol induced the expression of MMP-2 in keratinocytes, provide a possible explanation for cells’ increased cell migration and reduced inflammation.

Angiogenesis is a process regulated by complex mechanisms involving the proliferation and migration of endothelial cells, maturation and formation of a new basement membrane, and the creation of new blood vessels from existing ones [51,52,53]. Tie-2, a protein-tyrosine kinase cell surface receptor, plays an important role during angiogenesis. Studies have shown that both Tie-2 and its activated form, p-Tie2, are present on neonatal foreskin and adult skin epidermis [54]. Major ligands include angiopoietin-1 and -2 proteins with similar binding affinity and Tie-2 activation by Ang-1 in endothelial cells is able to enhance local vascularization by promoting blood vessels resistance and stability, and mediating angiogenesis by VEGF (Vascular endothelial growth factor) activating [55]. Lupeol treatment caused an increase in Tie-2 expression in human epidermal keratinocytes. Similarly, p-Tie2 was expressed in keratinocytes treated with lupeol. These findings support Tie-2 as an essential signaling component in lupeol-induced wound healing in human keratinocytes.

The epidermal layer of the skin is composed of keratinocytes in various stages of cell differentiation. After a damaging stimulus, keratinocytes are able to express different types of keratin proteins, different from those present in the healthy epidermis [56]. Cytokeratin 16 is rarely expressed under normal skin conditions, but may be found in oral mucosa and nails. This cytokeratin has a crucial role as a stimulatory keratin in skin diseases, especially in psoriasis, hypertrophic scars, other inflammatory conditions, and several types of cancers including squamous cell carcinomas [57,58]. It is important to note that reduced expression of keratin 16 contributes to the re-organization of important organelles of the cell, such as the cytoskeleton, which can directly affect the migration of keratinocyte [56]. Our study showed that lupeol treatment is capable of up- and down-regulating keratin 16 expression according to the concentration tested.

## 4. Materials and Methods

### 4.1. Plant Material, Extraction and Isolation of Lupeol

**Figure 11:**
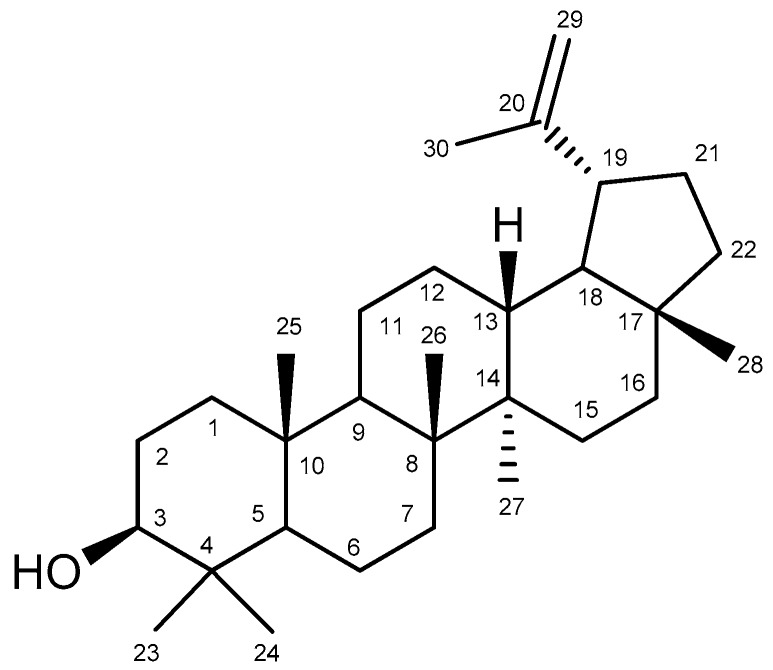
Chemical structure of lupeol.

*Bowdichia virgilioides* Kunth. (stem bark) was collected in December 2014 in the surroundings of Santa Rita, State of Paraíba, Brazil, a coastal area around the Atlantic Forest. A voucher specimen (*Agra et Góis 6243*) was deposited at the Herbarium Prof. Lauro Pires Xavier (JPB), and in the reference collection of the Laboratory of Pharmaceutical Technology from Federal University of Paraíba, João Pessoa, Brazil. Three kg of air-dried ground stem bark of *Bowdichia virgilioides* were exhaustively extracted with 95% alcohol solution. The extracted solution was filtered and the solvents were subjected to the evaporation method under reduced pressure with rotary evaporation (Solab, Piracicaba, São Paulo, Brazil) at 40 °C to obtain the final ethanolic extract (tHE, 250 g). The EtOHE was partitioned using solvents in increasing polarity (hexane, chloroform, and methanol). The hexane residue (49 g) was subjected to repeated washings with acetone under stirring followed by filtration. The solid obtained was recrystallized from chloroform and hexane, resulting in white crystals which were performed by analyzing ^1^H and ^13^C-NMR spectral data (Bruker, Billerica, MA, USA), compared with those published in the literature [59] and identified as lupeol (Figure 11) substance (3 g). The comparison of ^1^H and ^13^C-NMR data of lupeol isolated and ^13^C-NMR lupeol data in the literature [60] are presented at Table A1. A stock solution of lupeol (10 mg/mL) was prepared by dilution in dimethyl sulfoxide (DMSO) and alcohol. We defined as standard protocol for all treatments the final concentration of DMSO and alcohol 0.25 and 0.075%, respectively, which did not show cytotoxicity in previous reports [61].

### 4.2. Cell Isolation and Culture

Human primary epidermal keratinocytes and dermal fibroblasts were obtained from human neonatal foreskins following the standard protocols of the local ethics committee [62]. Keratinocytes were cultured in specific culture medium (Keratinocyte serum-free medium, K-SFM) and fibroblasts were cultured in Dublecco Modified Eagle’s Medium (DMEM), supplemented with 10% fetal bovine serum (FBS) and 100 units penicillin/streptomycin (Gibco-Life Technologies, Grand Island, NY, USA). All cells were plated and incubated at 37 °C in a 5% CO_2_ atmosphere and the culture media were changed three times per week. After reaching 70% confluency, the cells were trypsinised and seeded on specific plates for each analysis and incubated for 12 h prior to each experimental procedure.

### 4.3. Cell Proliferation Assay

The cell proliferation test was performed using the crystal violet assay. Cells were cultured to confluency and seeded in 96-well microplates (1 × 10^3^ cells/well) and after 24 h of incubation, the cells were treated in the absence or presence of lupeol at different concentrations (0.1, 1, 10, or 20 μg/mL). After 24 h of lupeol treatment, the cells were stained for 15 min with a solution of crystal violet (1 μg/mL) and subsequently washed with distilled water to remove any unbound dye residue. After complete drying of each well, 0.1% sodium dodecyl sulfate (SDS) buffer diluted in PBS (Phosphate-buffered saline) was added. The absorbance was read at 570 nm using a microplate reader.

### 4.4. Cytotoxicity Assay

Cell viability was assessed using MTT-based metabolic assay. Cells were cultured to confluency, and switched to DMEM with 0.1% BSA (Bovine serum albumin) for fibroblasts and adult keratinocyte growth medium without supplements for 24 h. MTT was added 4 h prior to completion of experiments. The absorbance was read at 570 nm using a microplate reader. Final results are expressed as percentages of controls.

### 4.5. In Vitro Migration (“Scratch”) Assay

Cell migration was assessed by “scratch” assay. Keratinocytes were seeded in 24-well plates and cultured to confluency. After 24 h incubation, a pretreatment with mitomycin (10 μg/mL) was performed to avoid any influence of cell proliferation [63]. Cells were scratched with a 100-μL blue plastic pipette tip after 2 h of pre-treatment with mitomycin, thereby creating a cell-free area measuring approximately 2 mm in width and photographed under phase-contrast microscopy (Olympus IX73 microscope, Olympus, Shinjuku, Tokyo, Japan). Cells were then immediately treated with lupeol at 0, 0.1, 1, 10 or 20 μg/mL concentrations and after 24 h new photographs were taken. The analysis was performed by counting cells that had moved from the initial area. The percentage of cell migration was calculated by the following formula: [number of moved cells after lupeol treatment/number of moved cells in the control condition] × 100.

### 4.6. In Vitro Wound Healing (“Scratch”) Assay

In vitro wound healing test was also evaluated using the scratch assay. Keratinocytes were cultured to confluency using 24-well plates and incubated for 24 h. The same procedure performed for scratch induction, as shown in Section 4.5., was also followed, except that mitomycin C was not added. Photographs were taken immediately after scratch and the cells were then treated under the same conditions performed in the cell migration test. After 24 h, new photographs were taken to analyze through the images the wound area remaining. The wound closure rate was determined by the initial and final wound areas during the wounding induction and wound closure percentage calculated by the following formula: [(initial − final)/initial] × 100.

### 4.7. Collagen Gel Contraction Assay

The collagen gel contractility test was performed using a collagen solution prepared following manufacturer’s protocols. Dermal fibroblasts were cultured to confluence before being embedded in a 3D collagen matrix to measure their long-term contractility. Fibroblasts were added to the collagen gel solution and seeded in 24-well plates at a density of 1.7 × 10^5^ cells/mL in each well. After a 1 h incubation to allow the collagen solution to gel, DMEM was added to each well and incubated for overnight. After the incubation, three washes with PBS were performed to remove serum, and DMEM containing 0.5% of FBS was added. Cells were then treated with lupeol at 0.1, 1, 10, or 20 μg/mL concentrations and gel attachment around the side wall of the well was carefully released with a spatula immediately after treatment. The contractile activity was evaluated after 48 h of treatments for each well using ImageJ 1.47i software.

### 4.8. Cell Lysate Preparation and Western Blot Analysis

Keratinocytes with required confluence were pre-incubated with 0.1–20 µg/mL lupeol for 24 h. After 24 h incubation, cells were then lysed in ice-cold lysis buffer supplemented with protease inhibitors and phosphate inhibitors and centrifuged at 12,000× *g* for 5 min. An aliquot of cell lysate was separated by 10% SDS-PAGE (Sodium dodecyl sulfate polyacrylamide gel electrophoresis) and transferred to a polyvinylidene difluoride membrane. After blocking in a 5% skim milk powder solution, the membranes were incubated with B-actin, phosphorylated (P) forms of p38 (Tyr182) and p38 (Santa Cruz Biotechnology, Santa Cruz, CA, USA) phospho-Akt (Ser473), Akt, phospho-p44/42 MAPK (Erk1/2) (Thr202/Tyr204), p44/42 MAPK (Erk1/2), Tie2 (D9D10), phospho-Tie2 (Tyr992), NF-κB-p65 (Ser536), MMP-2 (Cell Signaling Technology, Beverly, MA, USA), and Keratin 16 (Thermo Fisher Scientific, Waltham, MA, USA) overnight at 4 °C. The next day, all membranes were washed and incubated in secondary antibody for 1 h at room temperature. Proteins were detected by the ECL (electrochemiluminescence) detection system, (Amersham Biosciences, Little Chalfont, UK) and analyzed by ImageJ software.

### 4.9. Statistical Analysis

All data are expressed as the mean ± standard error of the mean (SEM). Statistical significance was performed by one-way ANOVA followed by Tukey’s test for all analyses. All data were examined using GraphPad Prism 6.0 software (GraphPad Software, Inc., La Jolla, CA, USA) and *p* < 0.05 was considered statistically significant.

## 5. Conclusions

In conclusion, the results of this study demonstrated the involvement of lupeol in the closure of skin wounds through the stimulation of the migration of keratinocytes and increased contraction of fibroblasts embedded in a collagen matrix. The underlying mechanism for the positive effect of lupeol on wound healing may involve the activation of PI3k/Akt and p38 MAPK, suppression of NF-κB signaling and Keratin 16, as well as the cyto-protective effects of MMP-2 and Tie-2. Although these results provide important information for lupeol in promoting wound healing in vitro, further in vivo and clinical studies are required to explore these and other pathways, as well as to develop lupeol as a therapeutic agent in the treatment of cutaneous wounds.

## Appendix A

**Table A1:** ^1^H and ^13^C-NMR (1D, 2D) spectra data for lupeol isolated from the *Bowdichia virgilioides* (400 MHz, CDCl_3_) and ^13^C-NMR data lupeol.

Lupeol (Isolated)						Lupeol (Literature)
C/H	δ^13^C/ppm	DEPT	δ^1^H/ppm (H Multiplicity)	HMBC	COSY	δ^13^C/ppm
1	38.75	CH_2_				38.9
2	27.48	CH_2_	1.89 (2H, m)		3.16 (H-3)	27.6
3	78.97	CH	3.16 (1H, m)		1.89 (H-2)	79.2
4	38.89	C				39.0
5	55.32	CH	0.66 (1H, m)		1.36 (H-6)	55.5
6	18.93	CH_2_	1.36 (2 H, m)			18.5
7	34.31	CH_2_				34.4
8	40.83	C				41.0
9	50.44	CH	1.24 (1H, sl)			50.6
10	37.17	C				37.3
11	20.97	CH_2_				21.1
12	25.13	CH_2_				25.3
13	38.05	CH				38.2
14	42.09	C				43.0
15	27.40	CH_2_				27.7
16	35.62	CH_2_				35.8
17	43.10	C				43.2
18	48.30	CH			2.37 (H-19)	48.5
19	48.02	CH	2.37 (1H, m)		1.36 (H-18)	
20	150.93	C				151.2
21	29.87	CH_2_				30.0
22	40.05	CH_2_				40.2
23	28.06	CH_3_	0.94 (3H, s)	55.32 (C-5); 78.97 (C-3); 15.49 (C-24)		28.2
24	15.49	CH_3_	0.74 (3H, s)	55.32 (C-5); 78.97 (C-3); 28.06 (C-23); 38.89 (C-4)		15.6
25	16.03	CH_3_	0.80 (3H, s)	50.44 (C-9); 55.32 (C-5)		16.2
26	16.20	CH_3_	1.01 (3H, s)	50.44 (C-9); 34.31 (C-7)		16.3
27	14.61	CH_3_	0.92 (3H, s)	27.40 (C-15)		14.7
28	18.08	CH_3_	0.77 (3H, s)	43.10 (C-17); 48.30 (C-18); 35.62 (C-16); 40.05 (C-22)		18.1
29	109.46	CH_2_	4.67 (1H, sl) 4.55 (1H, sl)	48.02 (C-19); 19.38 (C-30); 48.02 (C-19); 19.38 (C-30)	4.67 (H-29 a) 4.55 (H-29 b)	109.5
30	19.38	CH_3_	1.66 (3H, s)	109.46 (C-29); 151.93 (C-20); 48.02 (C-19)		19.5
